# Prediabetes and diabetes in a cohort of Qatari women screened for polycystic ovary syndrome

**DOI:** 10.1038/s41598-018-21987-6

**Published:** 2018-02-26

**Authors:** Soha R. Dargham, Abeer El Shewehy, Youssra Dakroury, Eric S. Kilpatrick, Stephen L. Atkin

**Affiliations:** 1 Infectious Disease Epidemiology Group, Weill Cornell Medicine, Doha, PO Box 24144 Qatar; 2 Research Faculty, Weill Cornell Medicine, Doha, PO Box 24144 Qatar; 3 Department of Pathology, Sidra Medical and Research Center, Doha, PO Box 26999 Qatar

## Abstract

Polycystic ovary syndrome (PCOS) is associated with an increased risk of *type 2 diabetes mellitus* (T2DM) but its association with prediabetes and T2DM is unknown in Qatar. *A cross sectional analysis of 3,017 Qatari subjects from the Qatar Biobank, identified 749 women aged 18–40 years, 720 of whom were assessed by the National Institute for Health (NIH) Guidelines for PCOS*. Prediabetes (HbA1c 5.7–6.4% and/or impaired fasting glucose (IFG*): fasting plasma glucose (FPG)* 100–125 mg/dL (5.6–6.9 mmol/L)), and T2DM (fasting plasma glucose > 125 mg/dL (≥7 mmol/L), and/or HbA1c ≥ 6.5%) were determined. *The prevalence of prediabetes was 10.6% and the prevalence of undiagnosed diabetes was found to be 4.0% in the total population. Overall, 12.1% of 720 women had PCOS, of whom FPG and HbA1c were available in 62 women with PCOS: 19.4% had prediabetes and 9.7% had diabetes. An adverse cardiovascular risk profile for IFG women compared to normal women was found*. Women with PCOS alone had a similar adverse cardiovascular profile as those with IFG alone and T2DM. Thus, the risk of prediabetes and diabetes is increased in Qatari women with PCOS, with an adverse cardiovascular risk profile similar to that seen in prediabetes and T2DM.

## Introduction

Polycystic ovary syndrome (PCOS) is the most common endocrine disorder among reproductive-aged women with a suggested prevalence of between 6% ((NIH) criteria) and 10% (Rotterdam and Androgen excess society guidelines)^1,2^. The irregular periods of PCOS lead to anovulatory infertility, and hirsutism and acne result from increased androgen levels^3,4^. An adverse cardiovascular profile is associated with PCOS, through a higher incidence of hypertension, an adverse lipid profile, and insulin resistance (IR)^5,6^. Obesity affects the majority of women with PCOS, and they have a higher prevalence of impaired glucose tolerance (IGT), reported as 4.7%, and a higher prevalence of type 2 diabetes (T2D), reported as 2.4 to 5.1% in cross-sectional studies, compared to a baseline prevalence of diabetes in non PCOS subjects of 1%^7–10^. A diagnosis of PCOS is said to confer a 5–10 fold increased risk of developing T2D and recent guidelines report IGT in obese and non obese US women with PCOS of 30–35% and 10–15%, respectively, with an additional 3–10% and 1–2%, respectively, having diabetes^1^. Two recent studies have suggested that obese but not non-obese PCOS were at increased risk of T2D^10,11^.

Prediabetes, also know as intermediate hyperglycemia or high risk for diabetes, is associated with the combination of insulin resistance and beta cell dysfunction, and it is reported that 5–10% of people with prediabetes convert to T2D though this may differ by population^12^. In addition to the increased cardiovascular risk that prediabetes confers, there is evidence that kidney and nerve damage may also be developing in this phase^12^, and it is therefore critical to address prediabetes at an early stage.

A small study of Qatari women estimated that the prevalence of PCOS was 18.3% and in a larger Qatari cohort was found to be 12.1% using NIH guidelines^13,14^, which could reflect a much higher incidence of prediabetes and T2D in these patients compared to a Caucasian population. This current study has used the national Qatar Biobank (QBB) to try and establish the incidence of T2D and prediabetes in PCOS Qatari women, with the hypothesis that if the prevalence of PCOS is increased in this population, then the prevalence of IGT and T2DM will also be increased proportionately.

## Materials and Methods

The QBB is a long-term project for the population of Qatar that is recruiting Qatari men and women aged 18–89 years, with the aim of subsequent follow up at 5 years. All subjects underwent detailed clinical, biochemical and genetic data collection approved by the QBB institutional review board (IRB) and by the Ministry of Health Qatar and all subjects gave their informed consent (http://www.qatarbiobank.org.qa). Subjects were recruited by advert and by word of mouth from those attending the QBB^13^. All participants entering into the QBB gave written informed consent for their data and samples to be used in research.

This study was a cross sectional study involving 749 PCOS women between the ages of 18-40 inclusive. A total of 720 (96.1%) had complete information to make a diagnosis of PCOS based on the NIH criteria of biochemical evidence of hyperandrogenemia (free androgen index > 4.5) and oligomenorrhea or amenorrhea. All had information to exclude other confounding diagnoses including thyroid function tests, prolactin, and 17 beta hydroxyprogesterone. All identified PCOS subjects had no documented concurrent illness and were not on any medication. All of the control women had regular menses, no biochemical hyperandrogenemia, no documented medical history, nor were taking any medications.

Height, weight, waist circumference and body mass index (BMI) were collected according to WHO guidelines^15^. Pulse Wave Velocity (PMV) was measured using VICORDER® PC 400 300E (SMT medical GmbH & Co. KG, Wuerzburg, Germany) as reported previously^13^.

All methods of analysis were performed in accordance with the relevant guidelines and regulations with appropriate quality control.

### Collection and analysis of blood samples

Blood samples were collected and immediately processed (within 5 min) and stored frozen at −80 °C pending analysis. General chemistry assays were analyzed by the National Centre for Cancer Care, Doha, Qatar, using a Roche Cobas analyzer (Roche Diagnostics, PO Box 50457, Indianapolis, USA), whilst immunoassays were performed for TSH, prolactin, insulin, testosterone, C reactive protein (CRP), DHEAS, and SHBG using an Abbott Architect analyzer (Abbott Laboratories, Abbott Park, Illinois, USA) using the manufacturer’s recommended protocol. The functional sensitivity of the testosterone assay was 0.49nmol/L with intra and interassay coefficients of variation for the assay of 10.0% and 11.3%, respectively. The free androgen index (FAI) was calculated as the total testosterone × 100/SHBG. Serum insulin was assayed using an Abbott Architect analyzer. The analytical sensitivity of the insulin assay was 2 μU/ml, the coefficient of variation was 6%, and there was no stated cross-reactivity with proinsulin. Plasma glucose was measured using a Roche Cobas analyzer. The coefficient of variation for the assay was 1.2% at a mean glucose value of 5.3 mmol/L during the study period. The insulin resistance was calculated using the HOMA method [HOMA-IR = (insulin × glucose)/22.5]. Data were supplied to Weill Cornell Medicine Qatar biostatistics (WCMQ) unit from the QBB in an anonymous coded manner that had been approved by the WCMQ IRB.

### Statistical analysis

The prevalence of PCOS among Qatari women from the general population or among groups at higher risk of developing PCOS is not known. Studies have estimated PCOS prevalence at about 10% among apparently healthy Caucasian women. To detect similar PCOS prevalence levels in this cohort with a precision level of 3%, assuming a standard normal variate of 1.96 for an alpha of 0.05, we estimated the minimum sample size needed would be 384 women. To detect a prevalence of T2D of 4% with 5% precision within that population, it would require the identification of 82 PCOS women for a significance level of 95% (type I error α = 0.05). Data trends were visually and statistically evaluated for normality. Non-parametric tests (Mann Whitney U) were applied on data that violated the assumptions of normality when tested using the Kolmogorov-Smirnov Test. Statistical analysis was performed using SPSS for Windows, version 24.0. All values are given as (mean ± SD) unless specified. All values are given as mean and 95% confidence interval (CI) unless specified.

## Results

Within the Qatar Biobank 749 of 3,017 Qatari subjects were women aged 18–40 years, of whom 720 had data to allow assessment under the NIH Guidelines for PCOS, from which 87 of 720 women fulfilled those guidelines (12.1%) for PCOS using a free androgen index greater than 4.5 ((testosterone/SHBG) × 100), and menstrual irregularity. Data for both fasting glucose and HbA1c was present in 676 subjects.

*The prevalence of prediabetes was found to be 10.6% overall (72/676 women): HbA1c 5.7–6.4% alone 17 subjects; IFG 100–125 mg/dL alone 43 subjects*; both HbA1c and IFG 12 subjects. Compared to normal women, those with IFG had a worse cardiovascular risk profile with a higher BMI (p < 0.001) higher waist hip ratio (P < 0.01), lower HDL cholesterol (p < 0.001), higher insulin levels and insulin resistance (<0.001), higher pulse wave velocity (p < 0.01), higher systolic and diastolic blood pressure (p < 0.01, and p < 0.03, respectively), and higher waist/hip ratios (p < 0.001). C reactive protein was also elevated (p < 0.04). The metabolic features of those with and without prediabetes are shown in Table 1. *Of the women with PCOS, 62 had a FPG and HbA1c measured: prediabetes was found in 19.4% (12/62)*, and T2DM in 9.7% (6/62). *IFG is associated with an adverse cardiometabolic profile that may be thought to be more adverse than that seen in PCOS. To address this issue, after the exclusion of prediabetes and T2DM from the PCOS cohort (44 women of the 62 noted above), this subset was compared to a group of non-PCOS women with prediabetes (60 women) that showed that the insulin resistance was higher (increased glucose and insulin) in non-PCOS women with prediabetes as expected (p < 0.01); however, in PCOS women it was seen that the HDL was lower and waist/hip ratio was higher in PCOS (p < 0.01) suggesting that those women with PCOS (without IFG) have a more adverse metabolic profile than normal women with IFG. Other metabolic parameters of systolic and diastolic blood pressure, endothelial function (Vicorder measurement), LDL and total cholesterol did not differ between them* (Table 2).

**Table 1:** Comparison of the metabolic features of women with (n = 72) and without (n = 604) prediabetes (HbA1c 5.7–6.4%; fasting glucose 100–125 mg/dl (5.6–6.9 mmol/l)). Mean (95% confidence intervals).

	Normal	Prediabetes	P-value
Age years	29.17 (28.66–29.68)	30.72 (28.93–32.51)	0.05
BMI	26.97 (26.44–27.50)	30.03 (28.07–32.00)	<0.001
Waist-Hip Ratio	0.74 (0.74–0.75)	0.78 (0.76–0.80)	0.001
Glucose mmol/L	4.74 (4.71–4.77)	5.91 (5.82–6.01)	<0.001
HBA 1C %	5.22 (5.19–5.25)	5.56 (5.41–5.71)	<0.001
Insulin pmol/L	10.64 (9.91–11.36)	27.91 (20.42–35.40)	<0.001
Insulin Resistance	2.30 (2.15–2.45)	10.69 (6.47–14.91)	<0.001
Total cholesterol mmol/L	4.76 (4.69–4.82)	4.77 (4.54–4.99)	0.83
High Density Lipoprotein mmol/L	1.55 (1.52–1.58)	1.43 (1.34–1.53)	<0.001
Low Density Lipoprotein mmol/L	2.76 (2.70–2.82)	2.81 (2.61–3.00)	0.74
C-Reactive Protein mmol/L	6.72 (6.29–7.15)	7.64 (6.26–9.02)	0.04
Testosterone mmol/L	1.21 (1.17–1.26)	1.21 (1.08–1.33)	0.92
Systolic blood pressure mmHg	104.5 (103.5–105.2)	108.7 (105.4–112.0)	0.01
Diastolic blood pressure mmHg	68.56 (67.81–69.31)	71.22 (68.70–73.74)	0.03
Vicorder - PulseWaveVelocity m/s	9.52 (9.36–9.68)	10.76 (9.36–12.16)	0.01

To convert values for for glucose to milligrams per decilitre, divide by 0.056. To convert values for insulin to picomoles per litre, multiply by 6. To convert values for cholesterol to milligrams per decilitre, divide by 0.0259. To convert values for triglycerides to milligrams per decilitre, divide by 0.0113.

**Table 2:** Comparison of the metabolic features of women with PCOS (without impaired fasting glucose or diabetes; n = 44) to those with prediabetes alone (without PCOS; n = 60). Mean (95% confidence intervals).

	PCOS	IFG (Pre-diabetes)	P-value
Age years	27.99 (26.68–29.29)	31.2 (29.27–33.14)	<0.01
BMI	30.52 (29.21–31.83)	28.80 (26.51–31.08)	0.07
Waist-Hip Ratio	0.79 (0.78–0.81)	0.76 (0.74–0.78)	<0.01
Glucose mmol/L	5.00 (4.79–5.21)	5.92 (5.81–6.04)	<0.001
HBA 1C %	5.55 (5.36–5.74)	5.51 (5.33–5.68)	0.95
Insulin pmol/L	15.52 (13.51–18.13)	28.81 (19.68–37.94)	0.016
Insulin Resistance	3.96 (3.10–4.83)	10.79 (5.57–16.01)	<0.01
Total cholesterol mmol/L	4.66 (4.50–4.82)	4.70 (4.47–4.92)	0.57
High Density Lipoprotein mmol/L	1.34 (1.27–1.40)	1.44 (1.33–1.55)	<0.01
Low Density Lipoprotein mmol/L	2.77 (2.63–2.92)	2.74 (2.54–2.94)	0.65
Testosterone nmol/L	1.74 (1.60–1.87)	1.09 (0.98–1.21)	<0.001
C-Reactive Protein mmol/L	8.23 (6.89–9.58)	7.68 (6.02–9.34)	0.43
Systolic blood pressure mmHg	109.26 (106.75–111.77)	106.70 (103.20–110.20)	0.31
Diastolic blood pressure mmHg	72.79 (71.09–74.49)	70.20 (67.68–72.72)	0.22
Vicorder - PulseWaveVelocity m/s	9.89 (9.46–10.32)	10.64 (8.94–12.35)	0.78

To convert values for glucose to milligrams per decilitre, divide by 0.056. To convert values for insulin to picomoles per litre, multiply by 6. To convert values for cholesterol to milligrams per decilitre, divide by 0.0259. To convert values for triglycerides to milligrams per decilitre, divide by 0.0113.

The prevalence of diabetes was found to be 4.0% overall (27/676), of whom 22.2% (6/27) had PCOS. No subject in the QBB had a FPG greater than 125 mg/dl with a concomitant HbA1c less than 6.5. Patients with diabetes were older (p < 0001) with a higher BMI and a greater waist/hip circumference (p < 0.01). Insulin, insulin resistance and Vicorder pulse wave velocity was greater in diabetes (p < 0.01), as was diastolic blood pressure (p < 0.04) (Table 3).

**Table 3:** Comparison of the metabolic features of women with (n = 27) and without (n = 608) type 2 diabetes. Mean (95% confidence intervals).

	HBA_1C_ ≤ 5.6	HBA_1C_ ≥ 6.5	P-value
Age years	28.82 (28.31–29.33)	34.40 (31.36–37.44)	<0.01
BMI	26.72 (26.21–27.23)	31.75 (27.03–36.48)	0.01
Waist-Hip Ratio	0.74 (0.74–0.75)	0.83 (0.76–0.91)	<0.01
Glucose mmol/L	4.80 (4.76–4.85)	12.66 (7.64–17.67)	<0.001
HBA 1C %	5.16 (5.13–5.18)	9.98 (8.39–11.57)	<0.001
Insulin pmol/L	11.78 (10.54–13.02)	76.69 (−55.09–208.47)	<0.01
Insulin Resistance	2.80 (2.40–3.20)	37.47 (−10.83–85.78)	<0.001
Total cholesterol mmol/L	4.75 (4.68–4.81)	5.18 (4.60–5.77)	0.052
High Density Lipoprotein mmol/L	1.56 (1.53–1.59)	1.43 (1.03–1.82)	0.264
Low Density Lipoprotein mmol/L	2.76 (2.69–2.82)	3.05 (2.71–3.39)	0.065
Systolic blood pressure mmHg	103.8 (103.0–104.7)	111.8 (102.67–120.9)	0.046
Diastolic blood pressure mmHg	67.90 (67.17–68.63)	73.10 (67.67–78.53)	0.064
C-Reactive Protein mmol/L	6.61 (6.18–7.04)	11.68 (4.81–18.55)	0.107
Vicorder - PulseWaveVelocity m/s	9.47 (9.31–6.94)	11.76 (10.24–13.28)	0.001

To convert values for glucose to milligrams per decilitre, divide by 0.056. To convert values for insulin to picomoles per litre, multiply by 6. To convert values for cholesterol to milligrams per decilitre, divide by 0.0259. To convert values for triglycerides to milligrams per decilitre, divide by 0.0113.

*It can be seen in* Table 1 *that there was a significant increase in the cardiometabolic risk indices with prediabetes for BMI, waist-hip ratio, glucose, HbA1c, insulin and insulin resistance, HDL, CRP, systolic and diastolic blood pressure, and pulse wave velocity. From* Table 2 *it can be seen that when IFG was compared to those with PCOS (without IFG), other than insulin and glucose for the IFG patients, all of the cardiometabolic risk indices became equivalent and indeed waist-hip ratio was higher and HDL was lower in PCOS, showing that the adverse metabolic profile in PCOS was equivalent or potentially worse than IFG alone. Similarly, in* Table 4 *when diabetes was compared to those with PCOS (without IFG or diabetes), other than insulin, glucose and pulse wave velocity for the diabetes patients, all of the cardiometabolic risk indices became equivalent though HDL was lower in PCOS, showing that the adverse metabolic profile in PCOS was similar to that seen for diabetes*.

**Table 4:** Comparison of the metabolic features of women with PCOS (without impaired fasting glucose or diabetes; n = 44) to those with diabetes (without PCOS; n = 27). Mean (95% confidence intervals).

	PCOS	Diabetes	P-value
Age years	27.99 (26.68–29.29)	34.40 (31.36–37.44)	<0.01
BMI	30.52 (29.21–31.83)	31.75 (27.03–36.48)	0.22
Waist-Hip Ratio	0.79 (0.78–0.81)	0.83 (0.76–0.91)	0.07
Glucose mmol/L	5.00 (4.79–5.21)	12.66 (7.64–17.67)	<0.001
HBA 1C %	5.55 (5.36–5.74)	9.98 (8.39–11.57)	<0.001
Insulin pmol/L	15.52 (13.51–18.13)	76.69 (−55.09–208.47)	<0.001
Insulin Resistance	3.96 (3.10–4.83)	37.47 (−10.83–85.78)	<0.001
Total cholesterol mmol/L	4.66 (4.50–4.82)	5.18 (4.60–5.77)	0.051
High Density Lipoprotein mmol/L	1.34 (1.27–1.40)	1.43 (1.03–1.82)	<0.01
Low Density Lipoprotein mmol/L	2.77 (2.63–2.92)	3.05 (2.71–3.39)	0.35
C-Reactive Protein mmol/L	8.23 (6.89–9.58)	11.68 (4.81–18.55)	0.13
Systolic blood pressure mmHg	109.26 (106.75–111.77)	111.8 (102.67–120.9)	0.16
Diastolic blood pressure mmHg	72.79 (71.09–74.49)	73.10 (67.67–78.53)	0.61
Vicorder - PulseWaveVelocity m/s	9.89 (9.46–10.32)	11.76 (10.24–13.28)	0.01

To convert values for glucose to milligrams per decilitre, divide by 0.056. To convert values for insulin to picomoles per litre, multiply by 6. To convert values for cholesterol to milligrams per decilitre, divide by 0.0259. To convert values for triglycerides to milligrams per decilitre, divide by 0.0113.

The calculated risk scores between groups using the Framingham equation^16^ (based on age, total cholesterol, HDL and systolic blood pressure, smoking and diabetes) were performed but did not differ between the PCOS, IFG and T2DM groups due to the young age of the population.

## Discussion

The overall prevalence of PCOS in Qatari women between the ages of 18 and 40 was 12.1% (using NIH criteria) and this was double that expected for a Caucasian population^2^. Therefore, it might be expected that the prevalence of prediabetes and diabetes in this population would be higher, thereby contributing to the very high prevalence of diabetes in Qatar of 23.3%^17^. This study shows that the overall prevalence of prediabetes by IFG and HbA1c in this cohort of women aged 18–40 years was 10.6%, whilst 19.4% of women with PCOS had prediabetes. *This would appear significantly higher in comparison to a study where the average age was 24 years and the prevalence of prediabetes determined by an oral glucose tolerance test (OGTT) was 7.1%*^18^, though in a large cohort aged 14–57 the prevalence of prediabetes determined by OGTT was not dissimilar at 23%. As an OGTT will detect more prediabetes than a FPG, then the prevalence of prediabetes in this Qatari population would likely be reported to be higher by this method^19^.

The diagnosis of prediabetes is important as it is a predictor of conversion to T2D and may depend on the ethnic population^19^. When subjects were followed up for 2.6 years, the conversion rate to IGT was reported as 11.5% with an annualized conversion rate at of 4.5%^18^, though other studies in differing populations suggest that the conversion rate to diabetes is 5–10%^20,21^. The conversion rate to diabetes is higher with IGT rather than IFG, as those with IGT may be close to the threshold of T2D whilst, by definition, IFG can only be close to the fasting criterion^19^. In a longitudinal study in Mauritius with a prevalence of diabetes similar to that of Qatar, the conversion rate of IFG to T2D was 4.6 to 6.7% per year^19^. It is recognized that those patients with prediabetes have an increased cardiovascular risk and it is recommended that active primary cardiovascular risk prevention should be initiated^22^. Patients with PCOS appear to have an increase in cardiovascular risk markers but it is unclear if this translates into increased or earlier cardiovascular disease^1,5^, and there are no long term prospective studies to quantify this. The data presented here raise significant concerns as the PCOS subjects had increased levels of the traditional cardiovascular risk indices similar to that of the IFG group, but with a lower HDL and a higher waist/hip ratio in the PCOS group that is associated with a more adverse cardiovascular profile. This suggests that Qatari women with PCOS have at least as high a risk of future cardiovascular disease as those with IFG. It should be noted, however, that in both groups the calculated cardiovascular risk was very low and difficult to determine due to the youth of the subjects. Compared to normal subjects, the PCOS and IFG groups showed an adverse metabolic phenotype with lower HDL levels and higher systolic and diastolic blood pressures^5^. CRP is a marker of inflammation and is associated with CVD^23^, and both PCOS and IFG groups had higher levels compared to normal women, though there was no difference for CRP between PCOS and IFG groups. Arterial stiffness correlates to cardiovascular risk^24^, perhaps through blood pressure variability^25^, and is measured by pulse wave velocity that was increased in both IFG and PCOS groups, contributing to the adverse cardiometabolic profile seen in PCOS and IFG.

The overall prevalence of diabetes in this cohort of women aged 18–40 was found to be 4% of whom 22% of the women with T2DM had PCOS*. The prevalence of diabetes in those women with PCOS was 9.7% that would appear significantly higher than the 5.1 and 5.7% reported previously*^8–10^, though obesity is a major factor with no excess diabetes risk in normal weight Caucasian women with PCOS^10^. The results reported here are, however, likely to be an underestimate as it has been shown in these PCOS women that the oral glucose tolerance test may be diagnostic of diabetes in the presence of a normal fasting blood glucose^1^. In addition, whilst the HbA1c is widely used to make a diagnosis of diabetes, its use in PCOS requires further validation given the concern that PCOS women may have T2D but with normal HbA1c^1^. Those with diabetes were older, with an adverse cardiovascular risk profile with a higher waist/hip ratio, higher diastolic blood pressure and higher pulse wave velocity measurement. *It is of concern that the cardiometabolic risk indices in those patients with diabetes were similar to those with PCOS apart from the higher pulse wave velocity in diabetes reflecting more endothelial dysfunction. However, although the metabolic risk factor of a low HDL was found in PCOS compared to diabetes, it is well recognized that the development of diabetes markedly increases the risk of cardiovascular mortality over metabolic syndrome indices alone*^26^. Currently, it is recommended that an OGTT is performed in women with PCOS for the diagnosis of T2D, particularly for those with a BMI over 30, gestational diabetes, a family history of diabetes or age greater than 40 years^1,27^ and perhaps repeated every second year^28^.

A major advantage of this study was its power to ascertain the prevalence of IFG using the combination of fasting plasma glucose and HbA1c. The major limitation of the study was that an oral glucose tolerance test was not performed as part of the recruitment into the biobank that may therefore have underestimated the number of subjects with T2D. Overall, the number of subjects with T2D were low in the PCOS group and a larger cohort in this ethnic group is needed to confirm the true prevalence of T2D. Recruitment of the QBB subjects may have been a source of bias as most were recruited from word of mouth; the heritability of PCOS is estimated to be 78% potentially leading to bias^29^. Some subjects were referred to QBB from secondary care with abnormal parameters of bone densitometry, frank hypertension or abnormal lipids; however, those parameters were not those of the population that was screened for this study.

In summary, for the overall population of 18–40 year old Qatari women, the initial prevalence of IFG was 10.6% and for diabetes 4%; however, 19.4% of women with PCOS had IFG, and 9.7% had T2D, with little difference in the metabolic parameters between PCOS, IFG and T2D, suggesting that Qatari women with PCOS have similar cardiovascular risk to those with hyperglycemia, and an active screening program of OGTTs should be implemented in this population in the Middle East and North African region.
